# Letter from Professor Martins

**Published:** 1840-08-29

**Authors:** 


### FOREIGN CORRESPONDENCE.

### LETTER FROM PROFESSOR MARTINS.

No. X.

*On the Use of Rhatany in the Treatment of Fissure of the Anus, and on some of the modes of employment of Oak-bark and Catechu.* (*Lecture of Professor* TROUSSEAU.)

PARIS, 6*th July*, 1840.

To the Editors of the Medical Examiner.

OF the various modes of application of rhatany, there is one entirely novel, which we owe to M. Bretonneau, of Tours. Accident led this skilful physician to the discovery; that is to say, one of those accidents which happen only to men capable of profiting by them. A lady, the wife of an officer, experienced severe pain in going to stool; her sufferings were such that she retained her fæces, and had only one alvine evacuation a week. M. Bretonneau thought that the constipation under which she laboured was owing to an amphoric dilatation of the lower portion of the rectum. In order to reduce the calibre of the intestine, and restore its contractile power, he prescribed injections of rhatany. At the expiration of fifteen days the lady was cured, and left the town of Tours. Two years after she wrote to M. Bretonneau that a lady, of about her own age, was affected with a *Fissure of the Anus*, the existence of which had been ascertained by a skilful surgeon. M. Bretonneau again recommended the use of injections of rhatany; this second patient was cured as rapidly as the first, to the great surprise of the attending surgeon. This second fact opened the eyes of M. Bretonneau; he recognised a fissure as the affection of the first patient as well as of the second,—and, for the last eight years, he has employed no other remedy than rhatany in the treatment of this disease. It was in 1809 that it was first described by Boyer; he defined it to be a spasmodic contraction of the sphincter, kept up by fissures. The treatment of Boyer consisted in incising deeply the fissure; the muscle was thus cut, and the fissure was cured, after a careful dressing, by means of tents introduced into the rectum. The operation was extremely painful, and the treatment kept the patient confined to bed for several weeks. M. Trousseau has tried the treatment of M. Bretonneau. A woman had been operated on by a surgeon for fissure of the anus; but the dressing by means of tents had not been done with care, and she entered the wards of M. Trousseau. She experienced the severest pains in going to stool; her evacuations were followed by palpitations which lasted for several hours, even when the stools were liquid. The first day, an injection merely of cold water was administered; when it was rejected, a quart injection was given of the following prescription:

℞. Extract. Krameriæ, ʒiss.

Tinct. " ʒss.

Aquæ Fontan, f.℥v.

M. f. sol.

The tincture of rhatany in this formula may be replaced by an equal dose of alcohol.

The patient retained this injection as long as possible. The next morning, another injection of pure water was administered, followed by two injections of rhatany, one in the morning, the other in the evening. This treatment was persisted in for a week with marked improvement; the pain, which formerly lasted for several hours after an evacuation, now continuing but a few minutes; after persevering in the treatment for another week, the cure was complete. For some time after, the use of simple injections was kept up, to avoid injuring a fresh cicatrix. This plan of treatment may serve as an example for those who wish to try it. It is usually at the end of a week that the improvement becomes decidedly marked, although sometimes the patient experiences relief from the first day. M. Trousseau has treated a patient, with a fissure an inch in length, suffering under an excessively painful diarrhœa. He was cured at the end of twenty-two days, although the cicatrix was torn during the treatment by the pipe of the syringe. Another patient had had a fissure for four months; after every evacuation she was in suffering for the rest of the day. At the expiration of eight days, her pain lasted no longer than a quarter of an hour; she left the hospital and returned at the end of two months, perfectly cured. A nurse had an excoriation at the posterior part of the anus; for eighteen hours after every stool she was in torment; after ten days' treatment, she was easy in three or four hours. She is still under treatment, and the cure will probably be longer than those of the others. M. Berard has treated with success two patients, each with a small fissure. M. Marjolin has cured one at the end of fifteen days. Some surgeons have proposed the extract of *monesia*, which has succeeded as probably all astringents would succeed. Catechu, tormentil, (Potentilla tormentilla,) bistort root, (Polygonum bistorta,) would undoubtedly be attended with equal success.

Catechu, the extract of the wood of the Mimosa catechu, the best of which comes from Bengal or Japan, has been proposed as a remedy for phthisis. M. Trousseau has made several trials of it. In decided phthisical cases, he has given as much as a drachm and a half a-day. The cough was somewhat allayed; the expectoration less copious; the diarrhœa ceased, and the sweating was somewhat less abundant, for some time; but soon all these symptoms reappeared, and it can hardly be affirmed that the lives of the patients were prolonged. Even in the commencement of phthisis, when the only apparent symptoms are expiration more prolonged, inspiration feebler, and one or two bloody expectorations, nothing more than temporary relief was obtained.

It is in chronic dyspepsia, accompanied with diarrhœa, and mucous secretions of the stomach like those of the bladder, that the extract of catechu is useful, in the dose of from ten to twenty grains a day. But the practitioner should always watch that the constipation which follows should not become prolonged, lest the presence of the fecal matter should develope a fresh inflammation of the mucous membrane, with which it is in contact.

In 1811, M. Goyet, director of the Veterinary School at Lyons, made some curious experiments upon the oak-bark, (Quercus robur.) During a month, he gave twenty pounds to a horse, and then knocked him on the head. At the autopsy, it was ascertained that the blood was more viscous, more consistent, and redder than usual, and the carcase of the horse remained two months exposed to the atmospheric vicissitudes, without putrefaction. At this time, putrid diseases were still held to, and the object of these experiments was to lead to some therapeutic treatment for them. Although we no longer believe in an analogy between the putrefaction which takes place after death, and the alterations of the blood which are supposed to exist in typhus fevers, it is, however, certain that astringents, applied upon the eschars which form upon the sacrum, in adynamic diseases, arrest their progress, and sometimes cause their cicatrization. These very eschars are an argument in favour of those who believe in an alteration of the blood in these diseases. For nothing is more certain than that they are produced with extreme facility in these patients, while they are very rare in individuals who remain sometimes forty days on their back, for fractures of the leg or thigh, or during the course of an acute disease.

